# The Clinical Varieties of Bright's Disease

**Published:** 1901-01-12

**Authors:** 


### THE CLINICAL VARIETIES OF BRIGHT'S DISEASE.

Addressing the Islington Medical Society, Dr. John Rose Bradford [1] recently described the several conditions which have been included under the term Bright's disease, and gave an outline of the views that have been held from time to time of their relation with one another. The relations of nephritis and congestion of the kidney to the first stage of Bright's disease are very close. Not only is there a great resemblance in the morbid anatomy of these conditions, but in their etiology as well, inasmuch as acute congestion of the kidney may unquestionably be produced by some toxic agents which also give rise to Bright's disease, and this is more especially the case in scarlet fever and other acute specific diseases. Some authorities, however, look upon these two terms as synonymous, and call all the cases Bright's disease; but there are clinical differences. The absence of dropsy, the earlier occurrence when it complicates a fever, and the more favourable progress in nephritis all tend to separate this condition from Bright's disease; and if we are to include all these cases under the heading of Bright's disease, which may perhaps be the simpler plan, we must, says Dr. Bradford, at least recognise that acute Bright has two forms, the course and the prognosis of which are very different.

In regard to chronic Bright's disease also two varieties must be recognised clinically, one with dropsy and the other without. These two varieties of chronic Bright's disease not only present different symptoms clinically, but the lesions found after death are notably different. To the form of disease where clinically dropsy is a prominent symptom the name "large white kidney" is usually applied. To the other form, characterised clinically by the absence of dropsy and the presence of grave nutritional and arterial changes, the term "contracted kidney," or "contracted white kidney," is perhaps most applicable. This condition, however, must be carefully distinguished from the true granular kidney which is not entitled to be regarded as a variety of Bright's disease, and is really a degenerative lesion associated with widespread arterial changes.

Summarising the matter, then, it may be said—(1) that we may recognise two forms of acute Bright's disease, one characterised not only by the well-known urinary changes, but also by the presence of dropsy; the other where dropsy is absent, a form the distinction between which and mere congestion of the kidney is by no means easy; (2) that there are at least two forms of chronic Bright's disease—one where the patient secretes a scanty, highly albuminous urine and becomes markedly dropsical, the course of the malady being chronic and death usually occurring either from the mere water-logging of the tissues, or from the development of inflammatory complications, or from chronic or subacute uræmia; the second, where the symptoms often run a latent course for an unknown period, and where the patient ultimately seeks advice on account of very vague symptoms of ill health, such as wasting, loss of strength, circulatory disturbance, or even where he does not seek advice until the onset of acute and fatal uræmia. In this form of the disease dropsy is absent, the urine is abundant and pale, and it contains a considerable quantity of albumen. It is to be remembered not only that chronic Bright's disease may be chronic from the outset, but also that the two varieties of chronic Bright are not necessarily different stages in one and the same morbid process, but represent rather the different effects of perhaps the same morbid process. It must be remembered also that outside and beyond all these forms of Bright's disease is the true granular kidney, which again may not come before us until the onset of uræmic symptoms, but as to the clinical differentiation of which Dr. Bradford did not enter into any details.

[1] Lancet, Jan. 5.

